# Quercetin Suppresses LCN2 and Restores Autophagy via the MAPK/ERK–mTOR Pathway in Bovine Endometrial Epithelial Cells and Attenuates Fibrotic Remodeling in LPS-Induced Mouse Endometritis

**DOI:** 10.3390/ijms27219865

**Published:** 2026-11-03

**Authors:** Ling Zhao, Xingyi Chen, Yi Zhou, Maimaiti Tuniyazi, Qinghua Shang, Jindong Gao, Mengdi Zhang, Hongwei Duan, Xiangguo Wang, Longfei Xiao

**Affiliations:** 1 College of Animal Science and Technology, Tarim University, Alar 843300, China; lingzhao1116@163.com (L.Z.);; 2 Animal Science and Technology College, Beijing University of Agriculture, Beijing 102206, China; chen-xingyi_xa@163.com (X.C.); 202330311015@bua.edu.cn (Y.Z.); 3 College of Veterinary Medicine, Anhui Agricultural University, Hefei 230036, China

**Keywords:** bovine endometritis, lipocalin-2, quercetin, autophagy, endometrial fibrosis, MAPK/ERK/mTOR

## Abstract

Bovine endometritis frequently progresses to endometrial fibrosis, a leading cause of implantation failure and infertility in dairy cows, yet the upstream events that disrupt endometrial autophagy during uterine inflammation remain poorly defined, and the anti-fibrotic target of quercetin (Que) in the bovine endometrium is unknown. We therefore combined data-independent acquisition (DIA) proteomics of healthy and fibrotic bovine endometria (*n* = 4 per group) with network pharmacology, molecular docking and a cellular thermal shift assay (CETSA), gain- and loss-of-function studies in TGF-β1-stimulated primary bovine endometrial epithelial cells (BEECs), and a 48 h LPS-induced mouse endometritis model in female C57BL/6 mice. LCN2 was markedly upregulated in fibrotic endometria and was prioritized among five candidate targets; docking predicted binding within the LCN2 pocket, and CETSA showed increased thermal stability of LCN2, consistent with target engagement. In BEECs, LCN2 overexpression activated MAPK/ERK–mTOR signaling, suppressed autophagic flux and promoted epithelial–mesenchymal transition (EMT), whereas LCN2 silencing reversed these phenotypes. Quercetin dose-dependently reduced LCN2 abundance and MAPK/ERK–mTOR phosphorylation, restored autophagic flux and reversed TGF-β1-induced EMT, and ERK inhibition abolished the LCN2-driven phenotype. In LPS-challenged mice, oral quercetin lowered uterine LCN2, recovered autophagy markers and reduced collagen deposition and endometrial damage. These preclinical findings associate LCN2 with impaired endometrial autophagy and EMT and suggest that quercetin may attenuate this cascade; binding affinity, LCN2 dependency and efficacy in cattle were not established and require validation in larger cohorts, purified-protein binding assays and chronic bovine models.

## 1. Introduction

Bovine endometritis is the most prevalent reproductive inflammatory disorder in postpartum dairy cows and a leading cause of impaired fertility worldwide [1,2]. Persistent endometrial inflammation damages the luminal epithelium and triggers progressive accumulation of extracellular matrix, resulting in fibrotic remodeling, intrauterine adhesions, embryo implantation failure and secondary infertility [3,4]. Pathological endometrial fibrosis causes irreversible intrauterine adhesions, impaired embryo implantation, and secondary infertility, drastically shortening the productive lifespan of dairy cows and reducing milk output, which imposes substantial annual economic losses for the global dairy industry [3,4]. Histologically, inflamed endometria undergo epithelial–mesenchymal transition (EMT): epithelial cells lose apical–basal polarity, and the epithelial marker E-cadherin, while acquiring mesenchymal markers N-cadherin and vimentin, and secrete abundant collagen to form persistent fibrous scars [5]. Therefore, suppressing EMT represents a promising therapeutic strategy to reverse endometrial fibrotic lesions. Clinical endometritis is defined by clinical signs of uterine inflammation, whereas fibrotic remodeling is a histological and molecular diagnosis; the two are related but are not equivalent, and this distinction is maintained throughout this study.

Autophagy is a lysosome-dependent homeostatic process that eliminates damaged organelles and misfolded proteins to sustain normal epithelial homeostasis [6]. Basal autophagy sustains endometrial epithelial cell (EEC) turnover and postpartum uterine tissue repair [7,8]. Accumulating evidence indicates that defective autophagy promotes EMT and fibrosis in pulmonary, hepatic and renal tissues [9,10,11], and recent studies in intrauterine adhesion models confirm that impaired autophagy drives endometrial EMT and collagen overproduction [12,13]. However, the upstream molecular mediator that disrupts autophagic homeostasis in the inflamed bovine endometrium has not been identified.

Lipocalin-2 (LCN2), also known as neutrophil gelatinase-associated lipocalin or uterocalin, is a pleiotropic protein of the lipocalin family that is strongly expressed in the uterine epithelium and markedly induced during uterine inflammation, postpartum involution and tissue remodeling [14,15]. Beyond its classical roles in iron sequestration and innate immunity, LCN2 has emerged as a pathogenic regulator of autophagy and EMT in several fibrotic disorders, including pulmonary fibrosis, steatohepatitis and retinal degeneration [16,17]. Whether inflammation-induced LCN2 acts as a bridging molecule that links autophagy impairment to EMT and fibrosis in the bovine endometrium remains unknown. Among the proteins upregulated in fibrotic bovine endometria in the present study, LCN2 was therefore selected for functional analysis, on the basis of its epithelial expression, its established links to autophagy and fibrotic remodeling, and its identification as a putative quercetin target by network pharmacology.

Quercetin is a natural flavonoid with well-documented antioxidant, anti-inflammatory and broad-spectrum anti-fibrotic activities [18,19]. Existing mechanistic studies of quercetin’s anti-fibrotic effects have concentrated on canonical TGF-β/Smad signaling or oxidative-stress pathways [20,21], and quercetin was recently reported to attenuate TGF-β1-induced fibrogenesis in human endometrial stromal cells through miR-145-mediated Smad2/3 inhibition [20]. In the kidney and myocardium, quercetin suppresses fibrosis partly by inhibiting mTORC1 signaling or by restoring autophagy [21,22]. Nevertheless, whether quercetin alleviates bovine endometrial fibrosis, and whether it acts through a direct functional target in endometrial epithelial cells, has not been explored. In cattle, recent work has begun to define the signaling pathways that drive endometrial fibrosis [23], but neither LCN2 nor quercetin has been examined in this context. In addition, the oral bioavailability of quercetin is low, and its metabolism in ruminants is uncharacterized, so any therapeutic application in dairy cows would require dedicated pharmacokinetic and formulation studies [24].

Despite this progress, three knowledge gaps persist in bovine endometrial fibrotic pathology and flavonoid therapy. First, the upstream molecular switch that disrupts autophagic homeostasis in the inflamed bovine endometrium is undefined, and whether inflammation-induced LCN2 bridges autophagy impairment to EMT-driven fibrosis is unknown. Second, no direct molecular interaction between quercetin and LCN2 has been validated, and the signaling cascade linking LCN2 to autophagy blockade in endometrial epithelial cells is uncharacterized. Third, integrated in vitro and in vivo evidence supporting quercetin as an anti-fibrotic agent for bovine endometritis is lacking. Guided by our DIA quantitative proteomic data showing markedly elevated LCN2 together with suppressed autophagy in fibrotic bovine endometria, we hypothesized that (1) inflammation-induced LCN2 hyperactivates the MAPK/ERK–mTOR axis, blocks autophagic flux and drives EMT-mediated endometrial fibrosis, and (2) quercetin suppresses LCN2 and interrupts this pathological cascade, thereby restoring autophagy and alleviating fibrotic injury. We addressed these hypotheses by integrating quantitative proteomics, network pharmacology, molecular docking, CETSA, genetic manipulation, autophagic-flux tracing, pathway-inhibition assays and an LPS-induced endometritis mouse model. These experiments were designed to test the stated hypotheses; the conclusions drawn below are restricted to the experimental systems used (bovine tissues, primary BEECs and an acute LPS-induced mouse model).

## 2. Results

### 2.1. Inflamed Bovine Endometrium Develops Severe Fibrosis Accompanied by Autophagy Deficiency and Elevated LCN2

**Figure 1:**
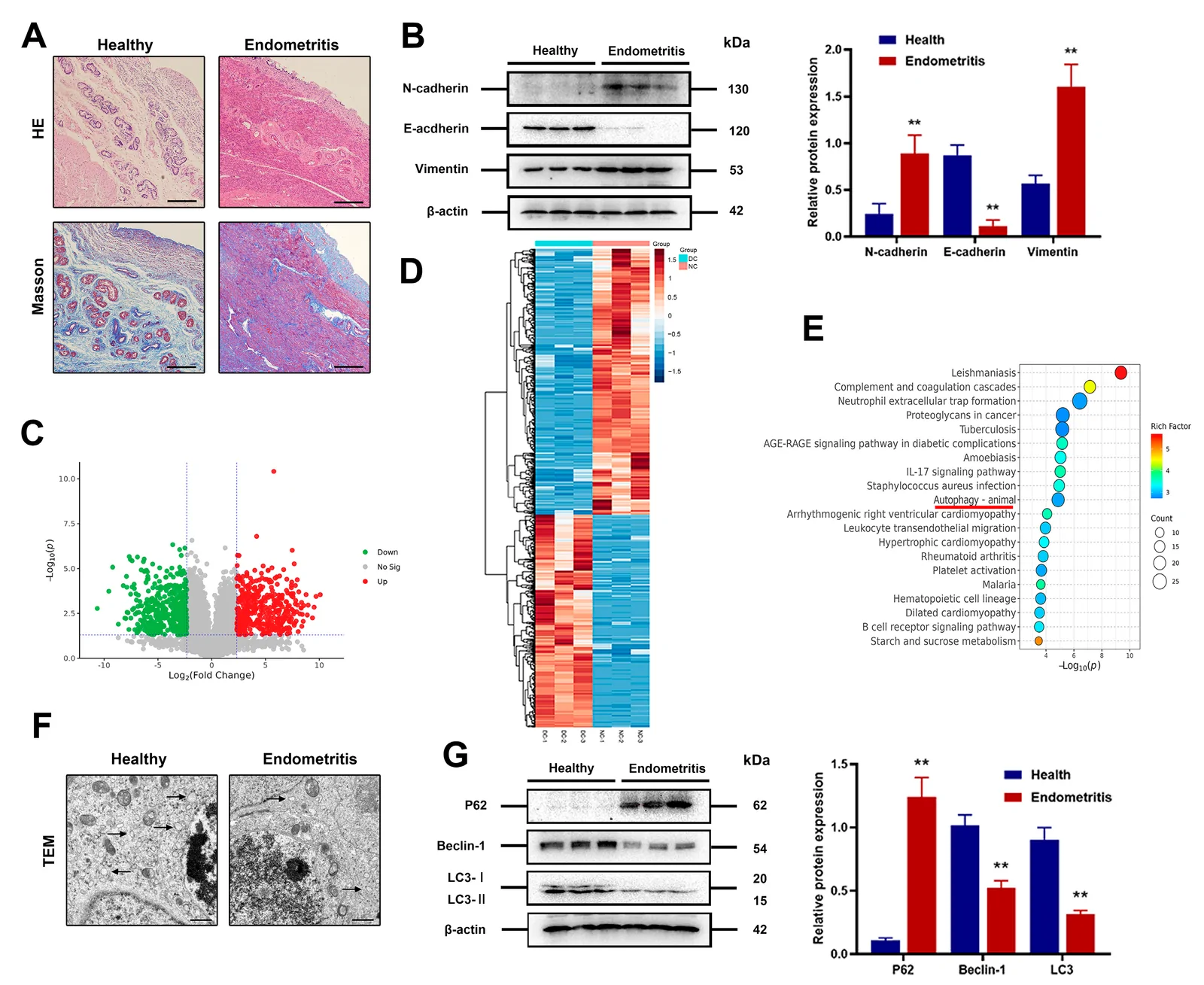
Autophagy defect and endometrial fibrosis in the inflamed endometrium. (**A**) H&E and Masson staining of the healthy and inflamed endometria. Scale bar = 100 μm. (**B**) Expression of the EMT-related proteins N-Cadherin, E-Cadherin, and Vimentin in the healthy and inflamed endometria. (**C**) Volcano plot of DEPs in the healthy and inflamed endometria based on DIA proteomics-sequence analysis. Green dots represent downregulated DEPs, and red dots represent upregulated DEPs (fold change ≥ 2, *p* < 0.05). (**D**) Heatmap of all DEPs in the healthy and inflamed endometria. (**E**) Top 20 pathways based on KEGG analysis of all DEPs. The Autophagy—animal pathway is significantly enriched among these pathways (Red line). (**F**) Ultrastructure of autophagy in healthy EECs and EECs with endometritis, observed by TEM. Scale bar = 500 nm. (**G**) Western blotting for expression of the autophagy-related proteins P62, Beclin-1 and LC3. β-actin is used as a loading control. Values are indicated as the mean ± SD; ** *p* < 0.01.

We first characterized the pathological differences between healthy and inflamed bovine endometrial tissues by histological staining (Figure 1A). H&E staining showed intact endometrial architecture and tightly arranged epithelial cells in healthy endometrium, whereas endometritis tissues displayed mucosal thinning and extensive epithelial shedding. Masson’s trichrome staining revealed regularly distributed collagen fibers alongside blood vessels and smooth muscle in healthy samples, while inflamed tissues showed irregular collagen deposition, increased neovascularization and expanded smooth-muscle layers, indicative of fibrotic remodeling (Figure 1A). Western blotting showed significant upregulation of the mesenchymal markers N-cadherin and vimentin and downregulation of the epithelial marker E-cadherin in endometritis tissues relative to healthy controls (Figure 1B), indicating EMT activation and fibrotic remodeling in the inflamed bovine endometrium.

To identify molecular pathways perturbed during endometrial fibrosis, DIA quantitative proteomics was performed on healthy and inflamed bovine endometria (*n* = 4 per group). With a threshold of fold change ≥ 2 and *p* < 0.05, 920 differentially expressed proteins (DEPs) were identified (Supplementary File S1). The volcano plot visualized upregulated (red) and downregulated (green) DEPs (Figure 1C), and hierarchical clustering clearly separated the healthy and endometritis groups (Figure 1D). KEGG pathway enrichment analysis identified autophagy among the top 20 significantly altered pathways (Figure 1E, Supplementary File S2). Transmission electron microscopy (TEM, scale bar = 500 nm) detected abundant autophagosomes in healthy EECs, whereas autophagosome abundance was markedly reduced in EECs from endometritis lesions (Figure 1F). Consistently, Western blotting showed that the autophagy initiators Beclin-1 and LC3 were downregulated while the autophagic substrate P62 accumulated in inflamed endometrium (Figure 1G), indicating impaired autophagy. Among the significantly upregulated DEPs, LCN2 was selected for functional validation as a candidate linking fibrosis and autophagy dysfunction, on the basis of its epithelial expression and its established roles in autophagy and fibrotic remodeling [14,15,16,17]. All quantitative data are expressed as mean ± SD; ** *p* < 0.01.

### 2.2. LCN2 Is Elevated in Fibrotic Endometria and Shows CETSA Evidence of Quercetin Target Engagement

**Figure 2:**
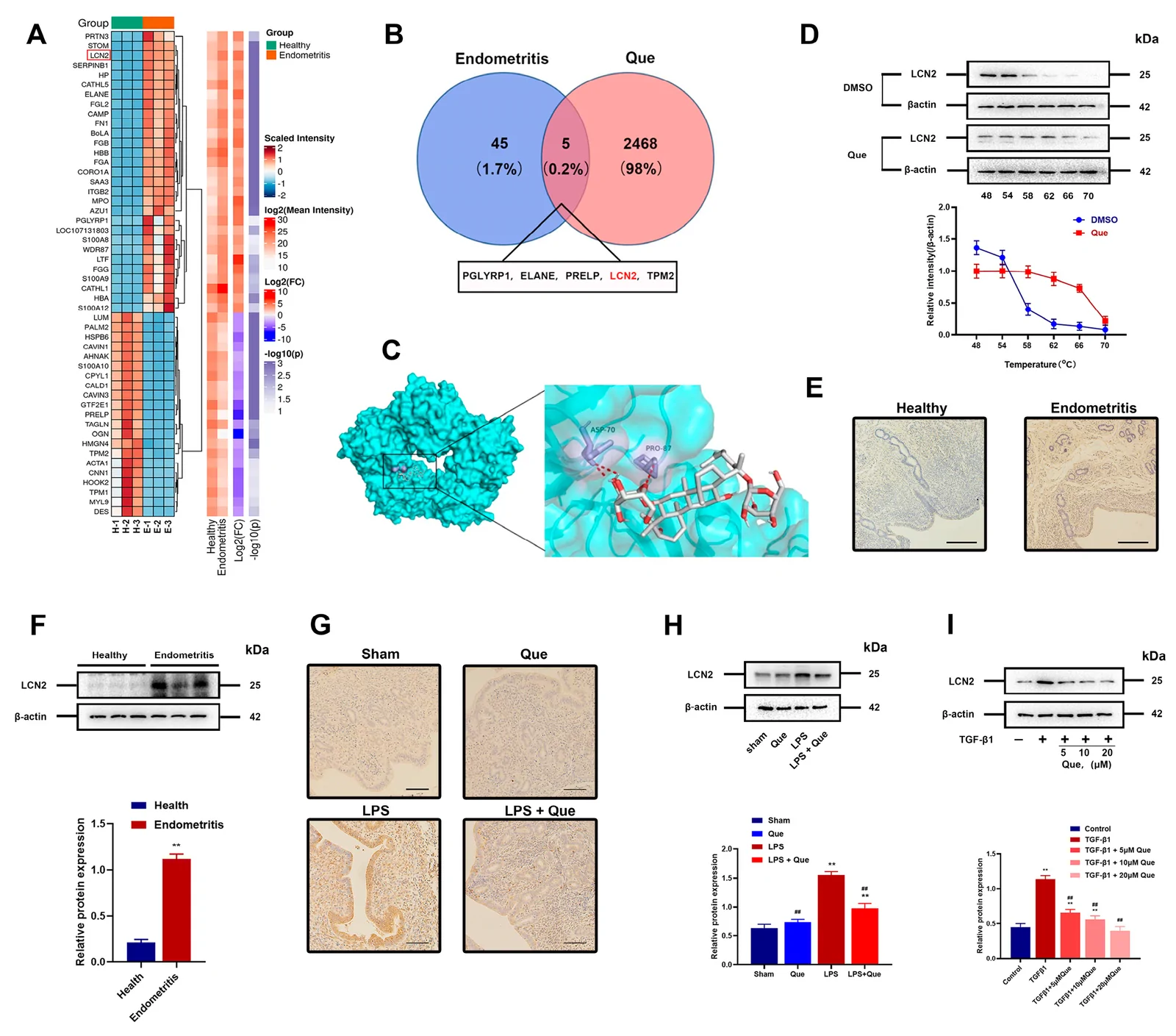
LCN2 is an upregulated candidate quercetin target. (**A**) Heatmap of the top 50 DEPs in the healthy and inflamed endometria. (**B**) Four quercetin target databases (TCMGeneDIT, BATMAN-TCM, TCMSP and TCMID) and the top 50 DEPs were used to predict the potential target genes of quercetin. (**C**) Docking of LCN2 with quercetin. Quercetin was predicted to interact with two amino acids, Asp70 and Pro87, adjacent to the LCN2 active site. (**D**) CETSA showing the effect of quercetin on the thermal stability of LCN2 (target-engagement assay). LCN2 expression was detected by Western blotting. (**E**) Distribution of LCN2 in the healthy and inflamed uteri. Scale bar = 100 µm. (**F**) Protein level of LCN2 in healthy and inflamed endometria detected by Western blotting. (**G**) Immunohistochemical staining of LCN2 in mouse uterus. Scale bar = 50 µm. (**H**) Protein level of LCN2 in mouse endometria measured by Western blotting. Values are presented as mean ± SD; ** *p* < 0.01 vs. the sham group; ## *p* < 0.01 vs. the model group. (**I**) Western blotting of LCN2 in BEECs treated with quercetin (at the indicated concentrations) in the presence of TGF-β1. Values are presented as mean ± SD; ** *p* < 0.01 vs. the control group; ## *p* < 0.01 vs. the TGF-β1 group.

Four compound-target databases (TCMGeneDIT, BATMAN-TCM, TCMSP and TCMID) were used to predict putative quercetin targets, yielding 2473 unique candidates after duplicate removal (Supplementary File S3). Intersection of these candidates with the top 50 upregulated DEPs generated five overlapping genes (Figure 2A,B), from which LCN2 was prioritized for the reasons described in Section 4.4. Molecular docking predicted favorable binding of quercetin within the LCN2 ligand-binding pocket, with hydrogen bonds to Asp70 and Pro87 (Figure 2C). CETSA provided independent evidence of target engagement: quercetin increased the thermal stability of LCN2 in BEEC lysates (Figure 2D). Because CETSA was performed in whole-cell lysates rather than with purified LCN2, these data support, but do not establish, a direct molecular interaction.

Immunohistochemical staining (scale bar = 100 μm) localized LCN2 predominantly to luminal and glandular epithelial cells of bovine endometrium, with weak expression in stroma (Figure 2E). Western blotting showed significantly higher LCN2 protein abundance in inflamed bovine endometrium than in healthy tissue (Figure 2F). We further detected LCN2 expression in the LPS-induced endometritis mouse model and TGF-β1-stimulated BEECs. Immunohistochemical staining of mouse uterine tissue (scale bar = 50 μm) showed elevated LCN2 expression in the LPS model group (Figure 2G). Western blot quantification showed increased LCN2 in uterine tissue of LPS-treated mice, while quercetin administration reduced LCN2 levels in vivo (Figure 2H). In vitro, TGF-β1 stimulation induced LCN2 overexpression in BEECs, and increasing concentrations of quercetin reduced LCN2 expression in a dose-dependent manner (Figure 2I). Collectively, these data show that LCN2 is elevated in inflamed endometrium and in the LPS-treated mouse uterus and that quercetin reduces LCN2 abundance in vitro and in vivo, consistent with target engagement; direct binding was not demonstrated. Quantitative data are expressed as mean ± SD; ** *p* < 0.01 vs. control/sham group; ## *p* < 0.01 vs. model/TGF-β1 group.

### 2.3. LCN2 Promotes MAPK/ERK–mTOR Signaling and Is Associated with Autophagy Suppression and EMT

**Figure 3:**
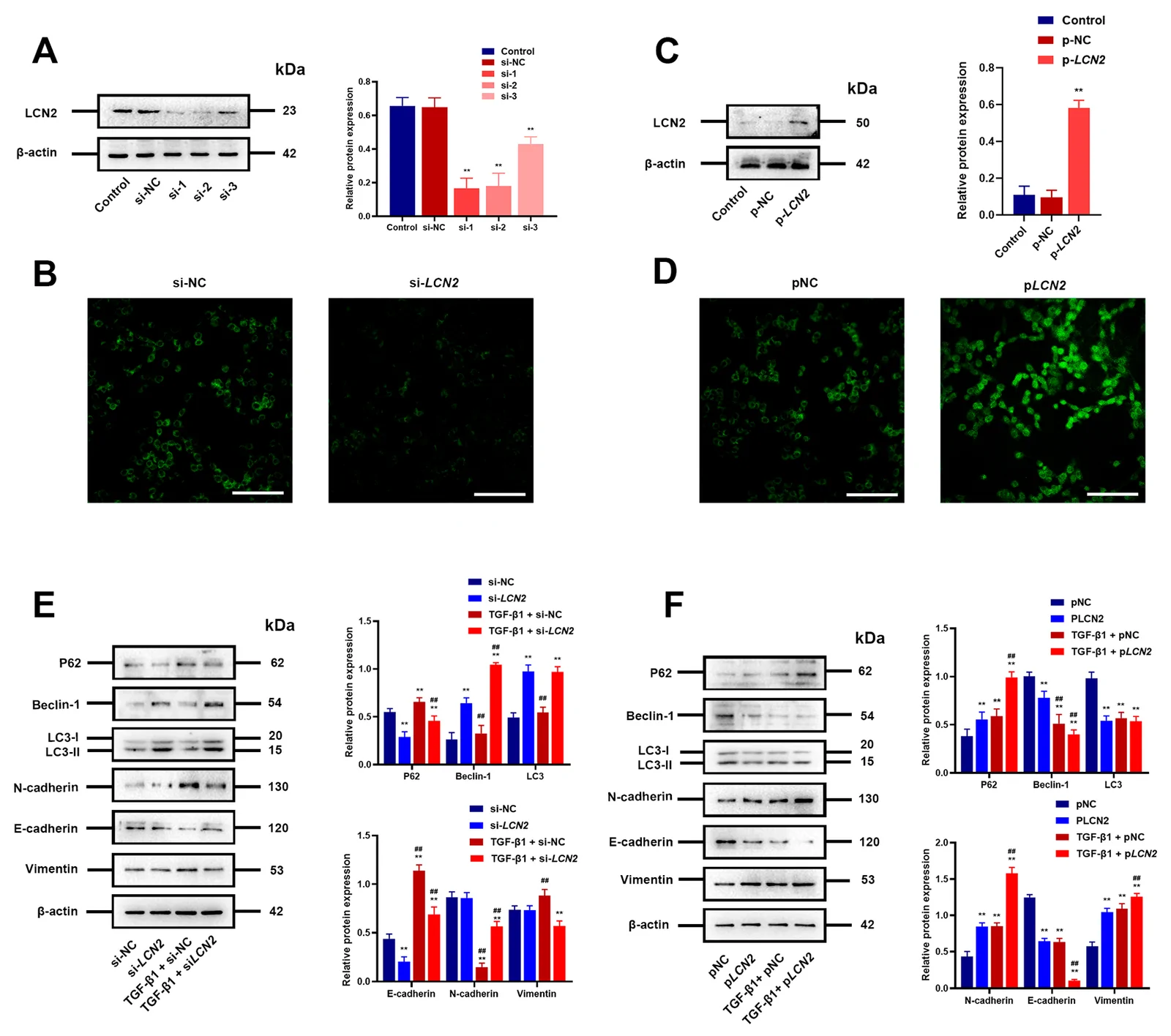
LCN2 regulates autophagy and epithelial–mesenchymal transition in BEECs. (**A**) Western blotting of LCN2 in BEECs transfected with three different LCN2 siRNAs (si-1, si-2 and si-3) or a negative control (si-NC) for 48 h. (**B**) Representative immunofluorescence images of BEECs transfected with si-NC or si-LCN2 for 48 h. Scale bar = 20 µm. (**C**) Western blotting of LCN2 in BEECs transfected with the LCN2 plasmid (pLCN2) or control plasmid (pNC) for 48 h. (**D**) Representative immunofluorescence images of BEECs transfected with pNC or pLCN2 for 48 h. Scale bar = 20 µm. (**E**) Western blotting of P62, Beclin-1, LC3, N-cadherin, E-cadherin and vimentin in BEECs transfected with si-NC and si-LCN2 in the presence of TGF-β1. (**F**) Western blotting of the same proteins in BEECs transfected with pNC and pLCN2 in the presence of TGF-β1. β-actin was used as a loading control. Values are presented as mean ± SD; ** *p* < 0.01 vs. the control group; ## *p* < 0.01 vs. the TGF-β1 group.

Loss- and gain-of-function experiments were performed to characterize the biological function of LCN2. Three independent LCN2 siRNAs (si-LCN2#1–#3) were transfected into BEECs for 48 h; si-LCN2#1 achieved the highest knockdown efficiency (Figure 3A), which was confirmed by immunofluorescence (scale bar = 20 µm; Figure 3B). Transfection of the LCN2 overexpression plasmid pLCN2 significantly increased LCN2 protein abundance compared with the empty vector pNC (Figure 3C), and immunofluorescence confirmed elevated LCN2 expression in pLCN2-transfected cells (scale bar = 20 µm; Figure 3D).

Under TGF-β1 stimulation, LCN2 silencing restored autophagy (increased Beclin-1 and LC3, decreased P62) and reversed EMT (elevated E-cadherin; reduced N-cadherin and vimentin) (Figure 3E). Conversely, LCN2 overexpression aggravated TGF-β1-induced autophagy suppression and mesenchymal transition (Figure 3F).

**Figure 4:**
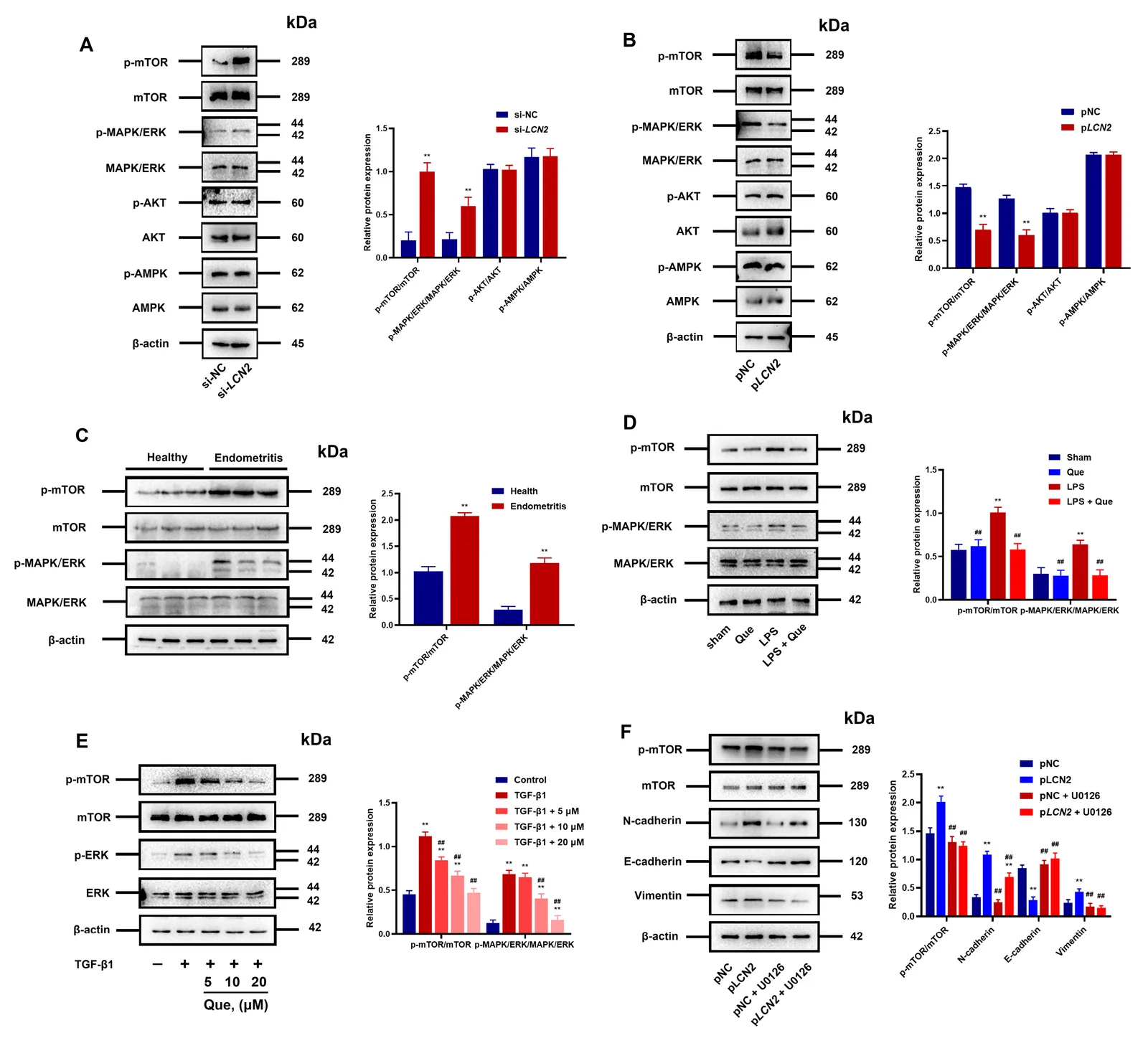
LCN2 regulates autophagy and EEC-EMT through the MAPK/ERK-mTOR signaling pathway. (**A**) The total and phosphorylated protein levels of mTOR, MAPK/ERK, AKT, and AMPK in BEECs after treatment with si-NC and si-LCN2 were detected by Western blotting. (**B**) The total and phosphorylated protein levels of mTOR, MAPK/ERK, AKT and AMPK in BEECs after treatment with pNC and pLCN2 were detected by Western blotting. (**C**) The total and phosphorylated protein levels of mTOR and MAPK/ERK in the healthy and inflamed endometria were detected by Western blotting. Values are indicated as the mean ± SD; ** *p* < 0.01. (**D**) The total and phosphorylated protein levels of mTOR and MAPK/ERK in mouse endometria were detected by Western blotting. The values are presented as mean ± SD; ** *p* < 0.01 vs. the sham group and ## *p* < 0.01 vs. the model group. (**E**) The total and phosphorylated protein levels of mTOR and MAPK/ERK in BEECs after treatment with quercetin (5, 10 or 20 µM) in the presence of TGF-β1 were detected by Western blotting. The values are presented as mean ± SD; ** *p* < 0.01 vs. the control group and ## *p* < 0.01 vs. the TGF-β1 treatment group. (**F**) The protein levels of p-mTOR, mTOR, N-cadherin, E-cadherin and Vimentin in BEECs (pretreated with 10 μM U0126) transfected with p-LCN2 for 48 h. The values are presented as mean ± SD; ** *p* < 0.01 vs. the p-NC group and ## *p* < 0.01 vs. the p-LCN2 group.

We next explored the downstream signaling mediators of LCN2. Western blot detection of kinase phosphorylation showed that LCN2 knockdown significantly reduced phosphorylation of MAPK/ERK and mTOR, with no obvious changes in phosphorylated AKT and AMPK (Figure 4A). Conversely, LCN2 overexpression enhanced P-MAPK/ERK and P-mTOR levels (Figure 4B). Consistent with cellular results, ex vivo inflamed bovine endometrial tissue exhibited elevated P-MAPK/ERK and P-mTOR compared with healthy endometrium (Figure 4C).

In addition, pharmacological intervention in the LPS mouse model showed that quercetin reduced mTOR and MAPK/ERK phosphorylation (Figure 4D). Upon TGF-β1 induction, quercetin was applied at gradient concentrations of 5, 10, and 20 μM. The results indicate that quercetin suppresses the mTOR-ERK signaling pathway in a dose-dependent manner at the cellular level (Figure 4E). To confirm the essential role of MAPK/ERK, we applied the ERK inhibitor U0126 in PLCN2-transfected BEECs. U0126 pretreatment blocked PLCN2-triggered mTOR hyperactivation and rescued abnormal EMT marker expression (Figure 4F). These results indicate that LCN2 activates the MAPK/ERK–mTOR cascade and is associated with autophagy suppression and EMT in BEECs. Quantitative data are expressed as mean ± SD; ** *p* < 0.01 vs. control/pNC group; ## *p* < 0.01 vs. TGF-β1/pLCN2 group.

### 2.4. Quercetin Restores Autophagic Flux and Reverses TGF-β1-Induced EMT in BEECs

**Figure 5:**
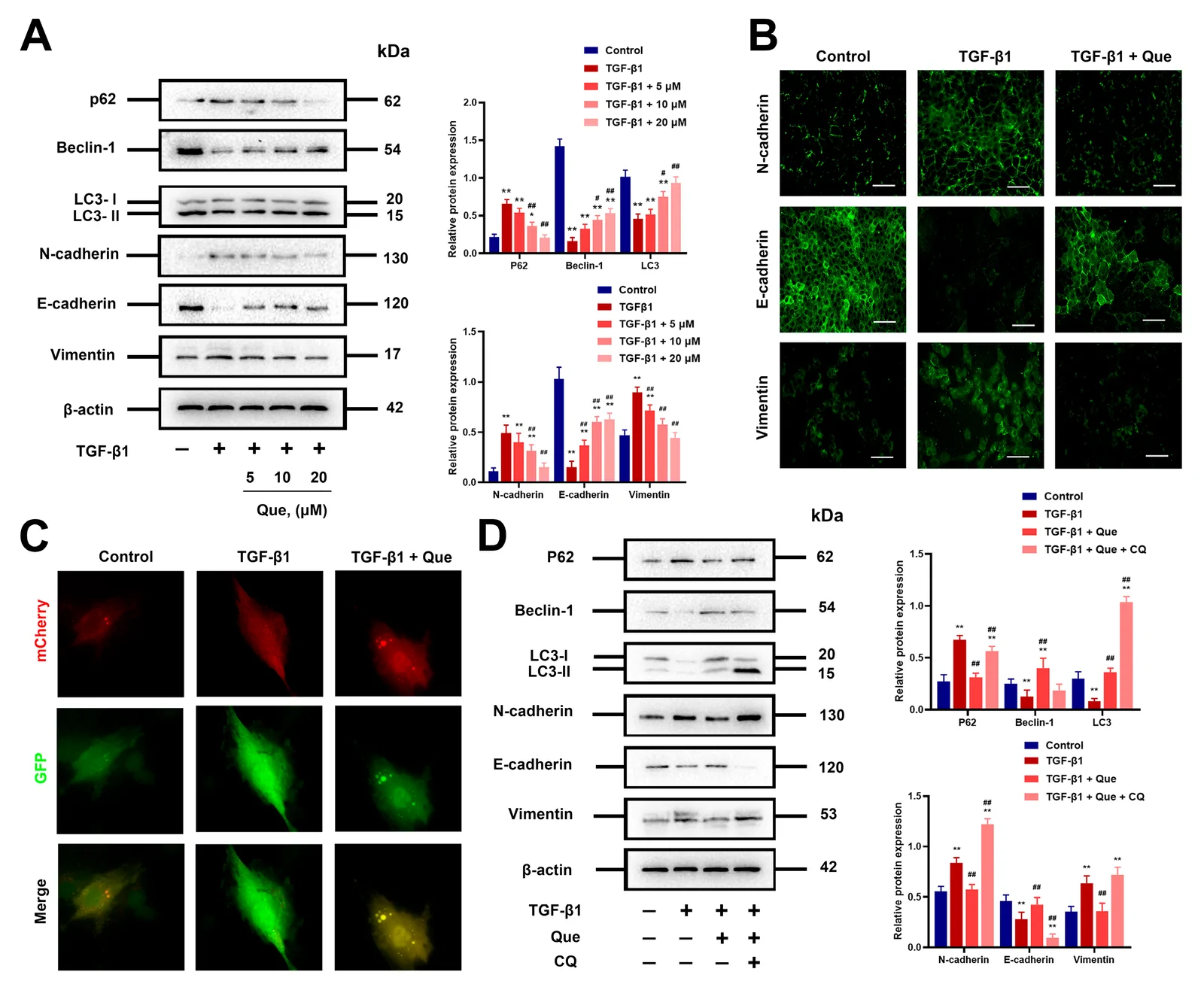
Activation of autophagy is associated with quercetin-induced inhibition of EEC-EMT in vitro. (**A**) BEECs were treated with TGF-β1 (10 ng/mL) and/or quercetin (at the indicated concentrations) for 48 h; Western blotting of P62, Beclin-1, LC3, N-cadherin, E-cadherin and vimentin. (**B**) Immunofluorescence analysis of N-cadherin, E-cadherin and vimentin in BEECs. Scale bar = 20 µm. (**C**) Autophagic flux was visualized by transfection of BEECs with the mRFP-GFP-LC3 reporter and quantification of autolysosomes. (**D**) BEECs were treated with TGF-β1 (10 ng/mL), quercetin (10 µM), Rapa (200 nM) and/or CQ (50 µM) for 48 h; Western blotting of P62, Beclin-1, LC3, N-cadherin, E-cadherin and vimentin. β-actin was used as a loading control. Values are presented as mean ± SD; * *p* < 0.05, ** *p* < 0.01 vs. the control group; # *p* < 0.05, ## *p* < 0.01 vs. the TGF-β1 group.

A TGF-β1 (10 ng/mL)-stimulated BEEC model was established to investigate the protective effects of quercetin. BEECs were treated with TGF-β1 and increasing concentrations of quercetin (5, 10 or 20 µM) for 48 h. Western blotting showed that TGF-β1 suppressed autophagy and induced EMT, whereas quercetin dose-dependently restored autophagy (increased Beclin-1 and LC3, decreased P62) and reversed EMT marker abnormalities (Figure 5A). Immunofluorescence staining (scale bar = 20 µm) confirmed that quercetin restored membrane-localized E-cadherin and reduced N-cadherin and vimentin fluorescence in TGF-β1-challenged BEECs (Figure 5B). Autophagic-flux monitoring with the mRFP-GFP-LC3 reporter showed fewer autophagosome and autolysosome puncta in TGF-β1-stimulated cells, and treatment with 20 µM quercetin effectively recovered autophagic vesicle formation (Figure 5C).

Pharmacological intervention experiments were performed to verify the causal relationship between autophagy activation and the anti-EMT effects of quercetin. BEECs were treated with TGF-β1, quercetin (10 µM), the autophagy agonist rapamycin (Rapa, 200 nM) and/or the autophagy inhibitor chloroquine (CQ, 50 µM) for 48 h. Western blotting showed that CQ abolished the inhibitory effect of quercetin on TGF-β1-induced EMT, whereas Rapa recapitulated the protective effect of quercetin by activating autophagy and suppressing EMT (Figure 5D). In addition, quercetin dose-dependently decreased the phosphorylation of MAPK/ERK and mTOR in TGF-β1-treated BEECs (Figure 4E).

### 2.5. Quercetin Attenuates LPS-Induced Inflammatory Endometrial Injury and Collagen Deposition in a Mouse Model

**Figure 6:**
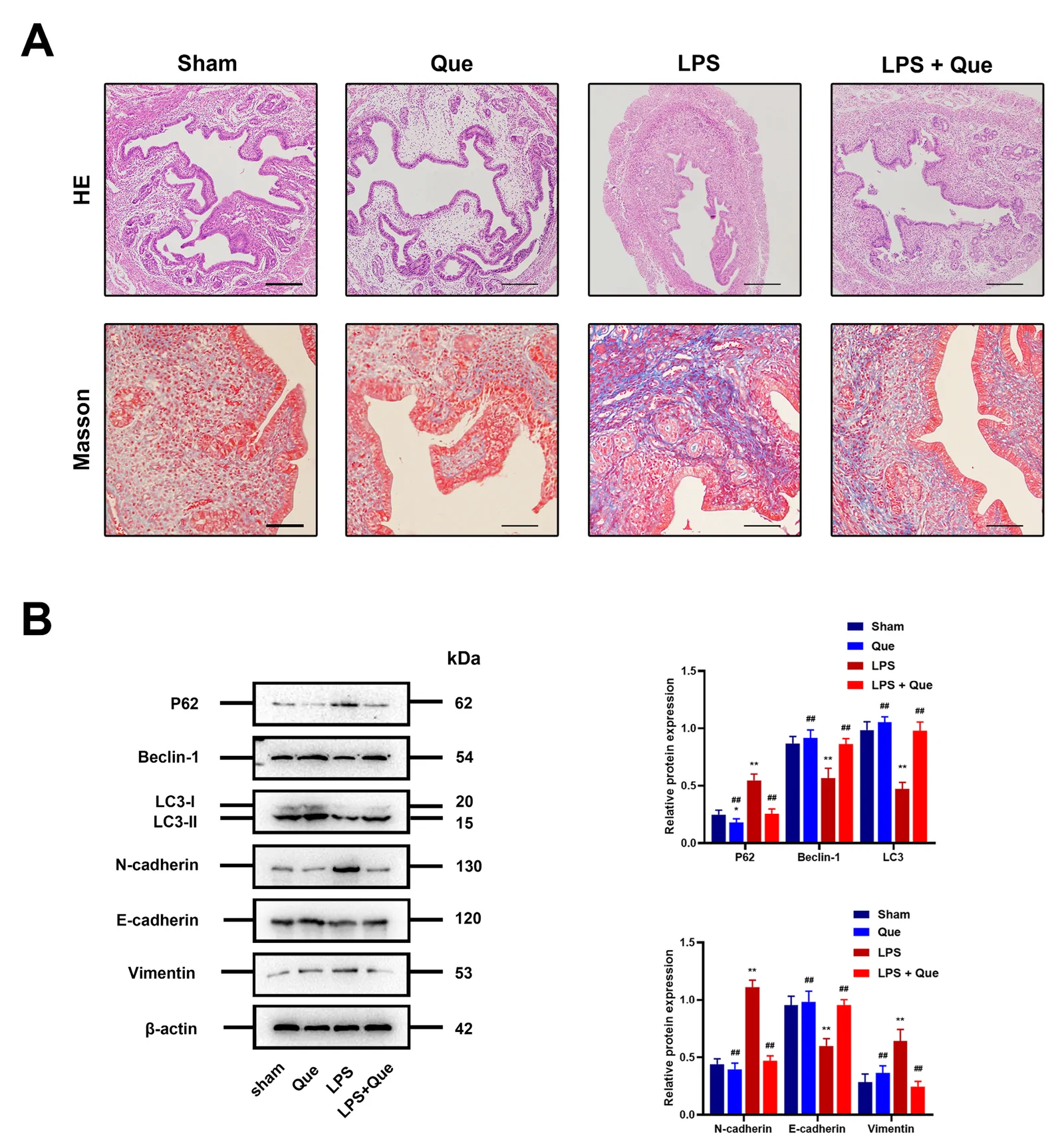
Quercetin improves autophagy and reverses endometrial fibrosis in the LPS-induced endometritis mouse model. Mice were divided into four groups (n = 6 per group): sham, quercetin (200 mg/kg), LPS, and LPS plus quercetin (200 mg/kg). (**A**) H&E staining (scale bar = 200 µm) and Masson’s trichrome staining (scale bar = 50 µm) of mouse uteri. (**B**) Western blotting of P62, Beclin-1, LC3, N-cadherin, E-cadherin and vimentin in mouse endometria. β-actin was used as a loading control. Values are presented as mean ± SD; * *p* < 0.05, ** *p* < 0.01 vs. the sham group; ## *p* < 0.01 vs. the model group.

An LPS-induced mouse endometritis model was used to test whether quercetin attenuates acute inflammatory endometrial injury in vivo. Mice were divided into four groups (n = 6 per group): sham, quercetin alone (200 mg/kg), LPS model, and LPS plus quercetin (200 mg/kg). H&E staining (scale bar = 200 µm) and Masson’s trichrome staining (scale bar = 50 µm) revealed severe endometrial gland atrophy and extensive collagen deposition in the LPS model group. Oral quercetin restored endometrial gland morphology and significantly reduced the fibrotic (collagen) area (Figure 6A). Western blotting of uterine tissues showed that LPS suppressed autophagy and triggered EMT, whereas quercetin reversed all LPS-induced changes in autophagy and EMT biomarkers (Figure 6B). Consistent with the in vitro findings, LPS exposure elevated uterine phospho-MAPK/ERK and phospho-mTOR levels in mice, and these increases were markedly attenuated by quercetin treatment (Figure 4D). These results indicate that quercetin attenuates LPS-induced endometrial injury and collagen deposition in mice; because this 48 h model reflects acute inflammation-associated remodeling rather than chronic fibrosis, the findings do not establish anti-fibrotic efficacy in cattle. Quantitative data are expressed as mean ± SD; * *p* < 0.05, ** *p* < 0.01 vs. the sham group; ## *p* < 0.01 vs. the model group.

### 2.6. Schematic Summary of the Proposed Mechanism

**Figure 7:**
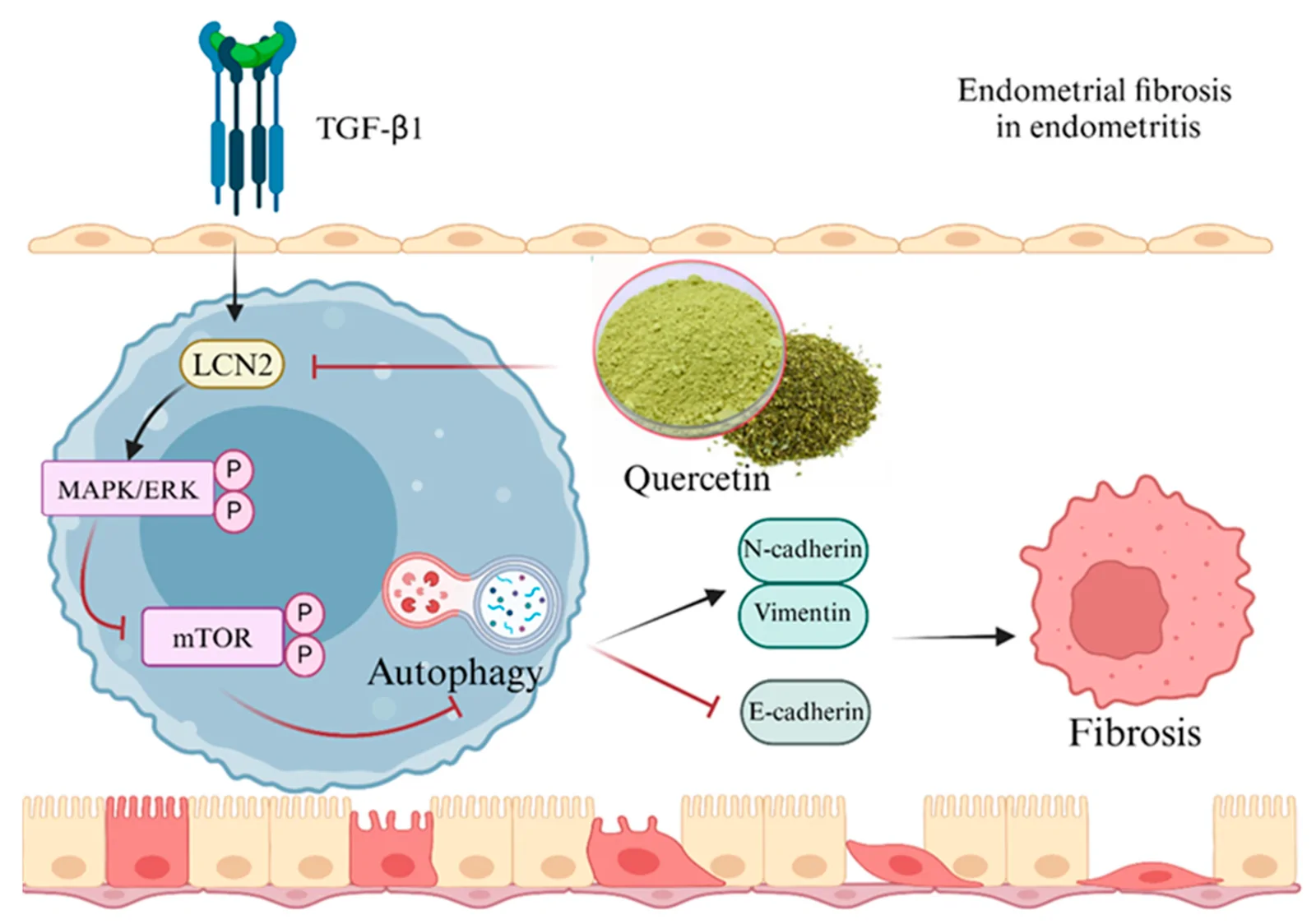
Proposed mechanisms underlying the effect of quercetin in alleviating endometrial epithelial–mesenchymal transition and fibrosis in bovine endometritis by suppressing the LCN2/MAPK–ERK–mTOR axis and restoring autophagy.

Collectively, the ex vivo bovine tissue, in vitro cellular and in vivo mouse model data are consistent with the working model illustrated in Figure 7. Inflammatory injury upregulates LCN2 in bovine endometrial epithelial cells. Accumulated LCN2 activates the MAPK/ERK–mTOR pathway, leading to autophagic-flux blockade, promotion of EEC EMT and progressive endometrial fibrosis. Quercetin suppresses LCN2, with docking and CETSA evidence of target engagement, reduces downstream MAPK/ERK–mTOR phosphorylation, restores autophagic activity, reverses EMT in BEECs and attenuates collagen deposition in the LPS-challenged mouse uterus (Figure 7).

## 3. Discussion

Bovine endometrial fibrosis driven by epithelial–mesenchymal transition is a major pathological cause of infertility and economic loss in high-yielding dairy herds [1,4]. In this study, we characterized a signaling cascade associated with endometrial fibrosis and the mechanism through which quercetin attenuates it. We show that inflammatory stimulation upregulates LCN2 in the bovine endometrium; accumulated LCN2 hyperactivates the MAPK/ERK–mTOR axis, blocks autophagic flux and drives EMT and extracellular-matrix deposition; and quercetin, which suppresses LCN2 abundance and shows CETSA evidence of target engagement, attenuates this cascade, restores epithelial autophagy, reverses EMT and alleviates endometrial fibrosis in vitro and in an LPS-challenged mouse model. This LCN2-centered model addresses the knowledge gaps outlined in the Introduction and provides preclinical, hypothesis-generating evidence that supports further investigation of quercetin; therapeutic efficacy in dairy cows was not evaluated.

Autophagy is an essential lysosomal homeostatic program that sustains normal endometrial epithelial structure and function under physiological conditions [7,8]. Consistent with prior research on intrauterine adhesion models [12,13], our ex vivo bovine tissue data revealed profound autophagic impairment in inflamed endometrium, manifested by reduced autophagosome abundance, suppressed Beclin-1 and LC3 expression, and accumulation of autophagic substrate P62. Pharmacological assays with chloroquine (autophagy inhibitor) and rapamycin (autophagy agonist) further supported a protective relationship between intact autophagy and suppression of EEC-EMT. Defective autophagy removes the intracellular quality control barrier that clears damaged organelles and profibrotic signaling intermediates, allowing epithelial cells to undergo sustained mesenchymal transformation and secrete excess collagen matrix [7]. While autophagy deficiency has been validated as a shared upstream driver of fibrosis across pulmonary, hepatic and renal tissues [9,10,11], the upstream molecular switch disrupting endometrial autophagy during bovine uterine inflammation remained uncharacterized prior to this work. Our proteomic screening and subsequent functional validation identify LCN2 as a candidate upstream mediator, addressing a gap in uterine fibrosis research.

LCN2 is a secreted protein of the lipocalin family that is constitutively expressed in the uterine epithelium and markedly induced during endometrial inflammation, postpartum involution and tissue remodeling [14,15]. Most mechanistic studies of LCN2 have focused on hepatic steatohepatitis, pulmonary fibrosis and retinal degeneration [16,17]. Notably, Gupta et al. showed that LCN2 binds ATG4B and interferes with LC3 lipidation, thereby reducing autophagic flux in retinal pigment epithelial cells [17]. Our data identify an additional, non-redundant mechanism in the bovine endometrium: LCN2 activates the MAPK/ERK–mTOR axis, and pharmacological blockade of MEK/ERK with U0126 abrogates both LCN2-driven mTOR activation and the fibrotic phenotype, indicating that ERK–mTOR signaling acts downstream of LCN2 in EECs. The observation that AKT and AMPK phosphorylation were unchanged further supports the selectivity of this axis. Whether LCN2 acts intracellularly or through its membrane receptors (e.g., SLC22A17/24p3R or MC4R) to engage ERK signaling in endometrial epithelial cells is an important question for future studies [15]. LCN2 also sequesters iron and can modulate ferroptosis and inflammatory signaling in other systems [14,25,26]; these extracellular and iron-related activities could contribute to the phenotypes observed here but were not dissected in the present study. Taken together, these observations associate LCN2 with the coordination of inflammation, autophagy suppression and EMT in bovine endometrial epithelial cells and identify LCN2 as a candidate target for further investigation; therapeutic validation in cattle has not been performed.

Quercetin possesses well-documented antioxidant, anti-inflammatory and anti-fibrotic activities [18,19]. Published mechanisms of quercetin-mediated anti-fibrosis are dominated by canonical TGF-β/Smad or oxidative-stress pathways [20,21], and, in the myocardium, quercetin promotes autophagy via the miR-223-3p/FOXO3 axis [22]. A distinct feature of this work is the observation that quercetin suppresses LCN2 in reproductive epithelial cells, in association with modulation of the MAPK/ERK–mTOR–autophagy cascade. Three lines of evidence—network-pharmacology prediction, molecular docking and CETSA—are consistent with a direct interaction between quercetin and LCN2, although binding affinity and specificity were not measured, and CETSA was performed in whole-cell lysates; dose-gradient experiments showed that quercetin reduces LCN2 abundance in a concentration-dependent manner, with corresponding reductions in MAPK/ERK and mTOR phosphorylation, restoration of autophagy and blockade of EMT. Quercetin also modulates many other molecular targets, including antioxidant enzymes, kinases and transporters, so the observed effects cannot be attributed to LCN2 alone [18,19,24]. The LCN2–autophagy axis described here may also be relevant to other forms of endometrial fibrosis, such as equine endometrosis and intrauterine adhesions (Asherman’s syndrome), in which TGF-β1-driven collagen deposition and impaired epithelial repair are central [27,28]; comparative studies are warranted. In vivo, the LPS-induced endometritis mouse model reproduced all in vitro phenotypes, and our results extend earlier evidence that quercetin-containing regimens attenuate uterine fibrosis in aged mice [29]. Quercetin is a natural dietary compound with anti-fibrotic activity in murine models [30]; however, its low oral bioavailability [24] and uncharacterized metabolism in ruminants mean that any application in dairy cows would require formulation and pharmacokinetic studies rather than direct extrapolation from the present results.

Several limitations warrant caution and guide future work. First, the in vivo validation relied on a 48 h LPS-induced mouse endometritis model, which recapitulates acute inflammatory injury rather than the chronic fibrotic remodeling seen in dairy cows; quantitative collagen measurement, longer-term models, primary bovine endometrial organoids and live-cow trials are needed before translational claims can be considered. Second, although docking and CETSA are consistent with an interaction between quercetin and LCN2, no quantitative binding measurements were performed, and CETSA was conducted in whole-cell lysates rather than with purified LCN2; binding affinity and specificity therefore remain undefined, and whether quercetin promotes LCN2 degradation or suppresses its transcription (e.g., via NF-κB) is unresolved. Future work should therefore include surface plasmon resonance or isothermal titration calorimetry with purified LCN2, together with analysis of LCN2 ubiquitination/proteasomal turnover and promoter activity. Third, the functional epistasis between quercetin and LCN2 (whether quercetin remains effective when LCN2 is silenced or overexpressed) was not tested, so the LCN2 dependence of the quercetin effect, although consistent with the present data, is not formally proven. Fourth, the bovine sample size (n = 4 per group) is small, was not determined by an a priori power calculation, and increases the risk of false-positive findings; the proteomic results should therefore be regarded as hypothesis-generating and confirmed in larger, independent cohorts. Quercetin bioavailability is also limited after oral administration, so intrauterine delivery, formulation and metabolite studies are warranted. Fifth, autophagic flux was assessed from LC3-II/LC3-I ratios, P62 abundance and mRFP-GFP-LC3 puncta, together with pharmacological blockade by chloroquine; puncta counting is semi-quantitative, and future work should apply guideline-recommended flux assays (e.g., LC3 turnover in the presence of lysosomal inhibitors with live-cell imaging or flow cytometry) [31]. Finally, LCN2 is a secreted protein, and its extracellular actions, including iron sequestration, receptor-mediated signaling (e.g., SLC22A17/24p3R) and neutrophil modulation, may contribute to the observed effects independently of intracellular ERK–mTOR signaling [14,25,26].

## 4. Materials and Methods

### 4.1. Animals and Tissue Sampling

Bovine tissue collection was approved by the Animal Ethics Committee of Tarim University and was performed in accordance with the Guidelines for the Ethical Treatment of Animals of the Ministry of Science and Technology of the People’s Republic of China. The uterine tissue sampling protocol was consistent with our previously published work [5]. Briefly, healthy Holstein cows and cows with clinical endometritis (n = 4 per group) were selected at a local slaughterhouse (Hebei, China); all clinical examinations described below were performed on the live animals before slaughter, and uteri were collected post-mortem immediately after slaughter. After the surrounding tissues were discarded, the uterine horns were opened longitudinally under sterile conditions on a clean bench. The endometrial tissue was mechanically isolated and divided into two parts: one part was used for isolation and culture of endometrial epithelial cells, and the other was cryopreserved at −80 °C. Residual tissue was fixed in 4% neutral paraformaldehyde (pH 7.4) for hematoxylin–eosin (HE) staining (G1120, Solarbio, Beijing, China), Masson’s trichrome staining (G1340, Solarbio) and immunohistochemistry [15]. Cows with endometritis were classified on the basis of clinical criteria; histological and molecular evidence of fibrotic remodeling was assessed subsequently, and the clinical diagnosis is therefore not equivalent to a histological diagnosis of fibrosis.

All cows had calved 5–6 weeks earlier and were undergoing postpartum uterine involution. Clinical examination and transrectal palpation were performed, and the cervical diameter was estimated per rectum. Real-time ultrasonographic imaging (Mindray M9, 5 MHz linear-array transducer, Shenzhen, China ) was used to evaluate the uterus and ovaries. Uterine swabs were obtained, and the vaginal discharge score was determined using methods described previously [32]. Table 1 summarizes the parity, body weight, body condition score and clinical criteria of the individual cows.

### 4.2. Data-Independent Acquisition (DIA)

Label-free quantitative proteomics was performed by Shanghai Bioprofile Technology Co., Ltd. (Shanghai, China). Total protein was extracted from healthy and inflamed bovine uterine tissue pellets using SDT lysis buffer. The digested peptides were desalted with peptide desalting spin columns, lyophilized under vacuum and quantified with a Nanodrop. Peptides were then separated on an Easy-nLC 1200 nano-flow chromatography system (Thermo Fisher Scientific, Waltham, MA, USA). Raw data were searched with MSFragger 3.4 software against the UniProt bovine proteome. Proteins with a fold change ≥ 2 (log2 fold change ≥ 1) and *p* < 0.05 were considered differentially expressed proteins (DEPs). The mass spectrometry proteomics data have been deposited to the ProteomeXchange Consortium via the iProX partner repository (accession number to be provided upon submission).

### 4.3. Establishment of Endometritis Mouse Model

The animal experiment was approved by the Institutional Animal Care and Use Committee of the College of Animal Science and Technology, Tarim University (Xinjiang, China; approval No. PA20251024028; approval date: 24 October 2025) and was conducted in accordance with the Guideline on the Humane Treatment of Laboratory Animals issued by the Ministry of Science and Technology of the People’s Republic of China.

Eight-week-old female C57BL/6 mice were provided by Beijing Vital River Laboratory Animal Technology Co., Ltd. (Beijing, China) and were acclimatized for 1 week on a basal diet. Mice were randomly assigned to four groups (*n* = 6 per group): (1) sham, (2) quercetin alone, (3) LPS model and (4) LPS plus quercetin. Endometritis was induced by intrauterine infusion of LPS (1 mg/kg, Sigma-Aldrich, Burlington, MA, USA; equal volume into each uterine horn) for 48 h; control mice received an equal volume of sterile saline under anesthesia. To test the effect of quercetin, mice received daily oral gavage of 100 µL quercetin (200 mg/kg/day, purity 99.80%, dissolved in ethanol and diluted with normal saline), a dose within the range reported to produce anti-inflammatory and anti-fibrotic effects in mice [30,33], beginning 2 days before LPS infusion; sham and model groups received equal volumes of saline. After treatment, mice were euthanized by cervical dislocation, and uterine tissues were collected for further analysis.

### 4.4. Potential Target Fishing and Molecular Docking

The 3D structure of quercetin was downloaded from PubChem (CID: 5280343). Putative quercetin target genes were predicted with four databases (TCMGeneDIT, BATMAN-TCM, TCMSP and TCMID), and the predicted targets were intersected with the top 50 upregulated DEPs using the Draw Venn Diagram tool (Venny 2.1.0). Five overlapping genes were obtained, and LCN2 was prioritized among them because it was one of the most strongly upregulated DEPs, is expressed in the endometrial epithelium, and has established roles in iron homeostasis, inflammation, autophagy and fibrotic remodeling [14,15,16,17,25]; the remaining four candidates were not examined further, which is acknowledged as a limitation. The 3D structures of LCN2 (PDB ID: 4MVK) and quercetin were prepared with AutoDockTools 1.5.4 (The Scripps Research Institute), removing surplus chains, water molecules and ligands before hydrogenation. Molecular docking was performed with AutoDock Vina, and the binding energy (BE) was used to evaluate binding affinity; a more negative BE indicates more favorable binding (the actual BE value is reported in the Section 2).

### 4.5. Cell Culture Plus Transfection

Primary bovine endometrial epithelial cells (BEECs) were isolated from healthy bovine endometrial tissues and cultured in DMEM/F12 (Hyclone, Logan, UT, USA) supplemented with 10% fetal bovine serum (FBS; Hyclone), 50 µg/mL streptomycin and 50 IU/mL penicillin G in a humidified incubator at 37 °C with 5% CO_2_. At approximately 80% confluence (after about 2 days), cells were seeded into 6-well plates (approximately 1.5 × 10^5^ cells/well) and cultured for 24 h before treatment.

To evaluate the anti-fibrotic effect of quercetin, BEECs were pre-incubated with quercetin (5, 10 or 20 µM; MedChemExpress, Monmouth Junction, NJ, USA) for 2 h and then stimulated with TGF-β1 (10 ng/mL; MedChemExpress, Shanghai, China) for 48 h. For pharmacological intervention, BEECs were incubated with or without TGF-β1 in the presence of 50 µM chloroquine (CQ, MedChemExpress), 200 nM rapamycin (Rapa, MedChemExpress) or 10 µM U0126 (MEK/ERK inhibitor, MedChemExpress) for 2 h before TGF-β1 treatment.

To examine the function of LCN2, BEECs at 70–90% confluence were transfected with three LCN2-specific small interfering RNAs (si-LCN2#1–#3), the LCN2 overexpression plasmid pcDNA3.1-CMV-LCN2 (pLCN2), or corresponding negative controls (si-NC; pNC) using Lipofectamine 3000 (Thermo Fisher, Waltham, MA, USA) according to the manufacturer’s instructions. Twenty-four hours after transfection, cells were stimulated with or without TGF-β1 for 48 h.

### 4.6. Cellular Thermal Shift Assay (CETSA)

BEECs overexpressing LCN2 (24 h after transfection) were harvested and lysed by freeze–thawing in liquid nitrogen, and the PBS-diluted lysates were divided into two aliquots that were incubated with quercetin (20 µM) or DMSO for 1 h at 37 °C. Each aliquot was then heated for 3 min at temperatures from 48 °C to 70 °C and cooled to room temperature. After centrifugation at 4 °C, the soluble fractions were analyzed by Western blotting.

### 4.7. Immunofluorescence Analysis

BEECs were seeded on coverslips in 6-well plates and cultured for 24 h, followed by 48 h of treatment with TGF-β1 (10 ng/mL) and/or quercetin (20 µM). Cells were washed three times with PBS, fixed in 4% paraformaldehyde for 15 min at room temperature and washed again.

After blocking with 5% BSA plus 0.1% Triton X-100 in PBS, cells were incubated overnight at 4 °C with primary antibodies against E-cadherin (1:100; 20874-1-AP, Proteintech, Rosemont, IL, USA), N-cadherin (1:100; 22018-1-AP, Proteintech) and vimentin (1:100; bs-0189R, Bioss, Woburn, MA, USA). After three washes, cells were incubated with FITC-conjugated goat anti-rabbit IgG (H+L) (1:200; TransGen, Beijing, China) for 45 min. Fluorescence images were captured with a fluorescence microscope.

### 4.8. Measurement of Autophagic Flux

To determine autophagic flux, BEECs were transfected with the mRFP-GFP-LC3 reporter plasmid (1 µL/mL; Addgene, Cambridge, MA, USA) and then treated with TGF-β1 (10 ng/mL) and/or quercetin (20 µM) for 48 h. Autophagosomes (yellow puncta) and autolysosomes (red-only puncta) were visualized by fluorescence microscopy; autophagosomes (yellow) and autolysosomes (red-only) were counted per cell and are reported as puncta per cell.

### 4.9. Western Blotting Assay

Tissues and cells were rinsed with ice-cold PBS and lysed in ice-cold RIPA buffer containing PMSF. Lysates were centrifuged at 12,000× *g* for 5 min at 4 °C, and protein concentrations were determined with a BCA Protein Assay Kit. Equal amounts of protein (20 µg) were separated by SDS-PAGE, transferred onto PVDF membranes (Millipore, Atlanta, GA, USA) and blocked with 5% skimmed milk in TBS/Tween for 1 h at room temperature. Membranes were incubated overnight at 4 °C with primary antibodies against N-cadherin (1:3000; 22018-1-AP, Proteintech), E-cadherin (1:3000; 20874-1-AP, Proteintech), vimentin (1:3000; bs-0756R, Bioss), P62 (1:1000; 18420-1-AP, Proteintech), LC3 (1:1000; 14600-1-AP, Proteintech), Beclin-1 (1:1000; 11306-1-AP, Proteintech), LCN2 (1:100; 30576-1-AP, Proteintech), mTOR (#2983, CST, Revesby, NSW, Australia), phospho-mTOR (Ser2448, #5536, CST), p44/42 MAPK (Erk1/2, #9102, CST), phospho-p44/42 MAPK (Thr202/Tyr204, #9101, CST), AKT (#4691, CST), phospho-AKT (Ser473, #4060, CST), AMPK (#2532, CST), phospho-AMPK (Ser485, #2537, CST) and β-actin (1:3000; bs-0061R, Bioss). After incubation with HRP-conjugated goat anti-rabbit secondary antibody (1:3000; bs-0295G-HRP, Bioss), signals were detected with ECL and quantified with ImageJ (v1.54g, NIH, Bethesda, MD, USA).

### 4.10. HE Staining, Masson Staining, and Immunohistochemical Staining

Paraffin-embedded tissue sections (5 µm) were stained with hematoxylin and eosin or Masson’s trichrome stain according to the manufacturer’s instructions (Solarbio). For immunohistochemistry, sections were incubated with anti-LCN2 antibody (1:100; 30576-1-AP, Proteintech) as described previously [5].

### 4.11. Transmission Electron Microscopy (TEM)

Glutaraldehyde (2.5%)-fixed tissues were post-fixed with 1% osmium tetroxide for 1 h at 4 °C, dehydrated, embedded in epoxy resin, sectioned and double-stained with uranyl acetate and lead citrate. Autophagic ultrastructure in bovine EECs was observed with a Hitachi H-7500 transmission electron microscope (Hitachi Ltd., Tokyo, Japan).

### 4.12. Data Analysis

All statistical analyses were performed with SPSS 19.0 (SPSS Inc., Chicago, IL, USA) or GraphPad Prism 9.5 (GraphPad Software, San Diego, CA, USA). Data are expressed as mean ± SD. Differences between two groups were analyzed by Student’s *t*-test, and comparisons among multiple groups were performed by one-way ANOVA followed by Tukey’s post hoc test. *p* < 0.05 was considered statistically significant. All in vitro experiments were repeated in at least three independent biological replicates; bovine tissues comprised four cows per group, and mouse experiments comprised six mice per group. Mice were randomly allocated to experimental groups. Investigators performing histological scoring and densitometric quantification were blinded to group allocation. No a priori power analysis was performed: the bovine sample size was dictated by the number of clinical cases available within the sampling period and by ethical and practical constraints. Mouse group sizes (n = 6 per group) were based on previous studies using the LPS-induced endometritis model and on animal-welfare considerations to minimize animal use; no formal a priori sample-size calculation was performed. The small number of animals limits statistical power and increases the risk of false-positive findings, and the proteomic results should therefore be regarded as hypothesis-generating.

## 5. Conclusions

In conclusion, this study associates inflammation-induced LCN2 with impaired endometrial autophagy and EMT in bovine endometritis and shows that quercetin reduces LCN2 abundance, inhibits MAPK/ERK–mTOR signaling and restores autophagic flux in bovine endometrial epithelial cells, with consistent effects in an acute LPS-induced mouse model. These findings extend the mechanistic understanding of flavonoid anti-fibrotic activity beyond canonical TGF-β signaling and support further evaluation of quercetin; however, a direct binding interaction, LCN2-dependent causality and efficacy in cattle were not demonstrated, so the results remain preclinical and require validation in larger cohorts, purified-protein binding assays and chronic bovine models.

## Figures and Tables

**Table 1:** Results of clinical examination.

Cow ID	T (°C)	Parity	Wt (kg) ^a^	BCS ^b^	BC ^c^	VDG ^d^	CD ^e^	U/S ^f^
1	38.3	2	625	4.00	Nil	0	<5 cm	CL, Fo
2	38.4	4	647	4.25	Nil ^g^	1	5 cm	CL ^h^, Fo ^i^
3	38.2	3	621	4.00	Nil	1	5 cm	CL, Fo
4	38.5	2	661	4.25	Nil	0	<5 cm	CL, Fo
5	39.6	3	624	3.75	E-c ^l^	3	>7.5 cm	UF, Fo
6	39.4	5	612	3.75	Mix	3	>7.5 cm	UF, Fo
7	39.5	2	587	3.50	Mix ^j^	3	>7.5 cm	UF ^k^, Fo
8	39.2	4	574	3.50	Mix	3	>7.5 cm	UF, Fo

^a^: Weight. ^b^: Body condition score on a scale of 1–5. ^c^: Bacterial culture. ^d^: Vulval discharge score on a scale of 0–3. ^e^: Cervical diameter. ^f^: Ultrasonography. ^g^: No colonies. ^h^: Corpus luteum. ^i^: Follicles. ^j^: Mixed colonies. ^k^: Uterine fluid. ^l^: *E. coli*.

## Data Availability

The data that support the findings of this study are available from the corresponding author upon reasonable request.

## Supplementary Materials

The following supporting information can be downloaded at: https://www.mdpi.com/article/10.3390/ijms27219865/s1.

## Abbreviations

The following abbreviations are used in this manuscript:

EECs	Endometrial epithelial cells
EMT	Epithelial–mesenchymal transition
TGF-β1	Transforming growth factor-β1
BEECs	Bovine endometrial epithelial cells
