# Supplementary material for: Quercetin Suppresses LCN2 and Restores Autophagy via the MAPK/ERK–mTOR Pathway in Bovine Endometrial Epithelial Cells and Attenuates Fibrotic Remodeling in LPS-Induced Mouse Endometritis
Source: Int J Mol Sci. 2026 Nov 3;27(21):9865. doi: 10.3390/ijms27219865 (PMC13763638; doi:10.3390/ijms27219865)
Supplement: Supplementary file 1 [file ijms-27-09865-s001.zip › Supplement file S4. Highlights.pdf]

Highlights

Inflamed bovine endometrium shows concurrent LCN2 upregulation, defective autophagy and aggravated EMT-driven fibrosis

LCN2 promotes endometrial epithelial fibrosis by activating the MAPK/ERK-mTOR pathway to block autophagic flux

Quercetin directly targets LCN2 to restore autophagy, reverse EMT and alleviate endometrial fibrosis both in vitro and in vivo

A new LCN2-centered therapeutic axis of quercetin is uncovered, supporting its application as a natural agent to treat dairy cow endometritis
