# Supplementary material for: Quercetin Suppresses LCN2 and Restores Autophagy via the MAPK/ERK–mTOR Pathway in Bovine Endometrial Epithelial Cells and Attenuates Fibrotic Remodeling in LPS-Induced Mouse Endometritis
Source: Int J Mol Sci. 2026 Nov 3;27(21):9865. doi: 10.3390/ijms27219865 (PMC13763638; doi:10.3390/ijms27219865)
Supplement: Supplementary file 1 [file ijms-27-09865-s001.zip › TABLE S1.pdf]

TABLE S1 Results of clinical examination

Cow ID	T (°C)	Parity	Wt (Kg)[a]	BCS[b]	BC[c]	VDG[d]	CD[e]	U/S[f]
1	38.3	2	625	4.00	Nil	0	<5 cm	CL, Fo
2	38.4	4	647	4.25	Nil[g]	1	<5 cm	CL[h] ,Fo[i]
3	38.2	3	621	4.00	Nil	1	<5 cm	CL, Fo
4	38.5	2	661	4.25	Nil	0	<5 cm	CL, Fo
5	39.6	3	624	3.75	E-c[l]	3	>7.5 cm	UF, Fo
6	39.4	5	612	3.75	Mix	3	>7.5 cm	UF, Fo
7	39.5	2	587	3.50	Mix[j]	3	>7.5 cm	UF[k], Fo
8	39.2	4	574	3.50	Mix	3	>7.5 cm	UF, Fo
